# Characterization of a lytic Salmonella phage GF04 within the *Jerseyvirus* lineage and identification of *yjiK*_2 as a candidate phage receptor

**DOI:** 10.3389/fmicb.2026.1828982

**Published:** 2026-05-14

**Authors:** Gabriel H. Fares, Salman A. Almashtoub, Tasnime A. Abdo Ahmad, Elias D. Antoun, May N. Abi-Mosleh, Sara Barada, Ahmad Turk, Ghassan M. Matar, Esber S. Saba

**Affiliations:** 1 Department of Experimental Pathology, Immunology, and Microbiology, Faculty of Medicine, American University of Beirut, Beirut, Lebanon; 2 Department of Biomedical Engineering, Maroun Semaan Faculty of Engineering and Architecture, American University of Beirut, Beirut, Lebanon; 3 Center of Infectious Diseases Research, Faculty of Medicine, American University of Beirut, Beirut, Lebanon; 4 WHO CC for Reference & Research on Bacterial Pathogens, Faculty of Medicine, American University of Beirut, Beirut, Lebanon

**Keywords:** bacteriophage, phage receptor, phage resistance, phage therapy, *Salmonella enteritidis*

## Abstract

**Introduction:**

*Salmonella enterica* is a major antimicrobial-resistant pathogen recognized by the World Health Organization, with high infection and mortality rates largely driven by antibiotic overuse. Bacteriophage therapy has emerged as a promising alternative due to phages’ high specificity, self-replication at infection sites, and safety when properly selected; however, its success depends on understanding phage–host interactions, particularly receptor recognition and resistance mechanisms.

**Methods:**

Salmonella phage GF04 was isolated from sewage using *S. enterica* serovar Enteritidis strain SAL271 as host and purified through successive plaque isolation. Phage characterization included adsorption kinetics, one-step growth curve analysis, bacteriolytic activity across multiple MOIs, and host range determination using efficiency of plating (EOP) on 19 clinical isolates. Whole genome sequencing and annotation were performed to assess taxonomic placement and the presence of virulence, antimicrobial resistance, and lysogeny-associated genes. A spontaneous phage-resistant derivative was isolated following phage exposure and analyzed by comparative whole genome sequencing and adsorption assays. Structural effects of identified mutations were evaluated using AlphaFold modeling.

**Results:**

GF04 forms clear plaques and exhibits rapid adsorption, with most virions attaching within 5 minutes. One-step growth analysis revealed a ~45-min latent period and a burst size of ~103 PFU per infected cell, indicating efficient replication. GF04 suppressed bacterial growth across multiple multiplicities of infection and demonstrated a relatively broad host range within the tested isolates, with clear strain-dependent variability, infecting 14 of 19 *S. Enteritidis* isolates as determined by efficiency of plating (EOP) analysis. EOP values ranged from no detectable infection to high efficiency (up to 0.8 relative to the reference host). Whole genome sequence analysis confirmed the absence of detectable lysogeny, virulence, or antimicrobial resistance genes, and phylogenetic analyses demonstrated that GF04 is a new species within the *Jerseyvirus* lineage (family *Sarkviridae*). Furthermore, a spontaneous phage-resistant variant of SAL271 exhibited a complete loss of phage adsorption compared to the wild-type strain. Comparative genomic analysis subsequently identified a fixed missense mutation (P128L) in the predicted membrane-associated gene *yjiK_2*. Structural modeling suggested that this mutation induces localized conformational changes potentially affecting receptor accessibility.

**Discussion:**

Comparative analysis of the resistant derivative implicates the mutation in *yjiK_2* as the most likely genetic basis of resistance, potentially underlying the observed loss of phage adsorption. This suggests that *yjiK_2* may encode or contribute to a receptor-associated determinant involved in GF04 adsorption. Together, these findings support the lytic potential and preliminary therapeutic relevance of GF04 while identifying *yjiK_2* as a candidate phage-resistance determinant that warrants further functional validation.

## 1 Introduction

*Salmonella* bacteria have emerged as a major concern due to their ability to persist in various niches, including poultry, eggs, meat, dairy products, fresh produce, and a variety of ready-to-eat products (Lopez-Garcia et al., 2023). Human infection typically occurs through the consumption of undercooked or cross-contaminated food products, especially poultry and eggs (Park et al., 2014). *S. enterica* subsp. *enterica* accounts for approximately 99% of human salmonellosis cases and includes non-typhoidal serovars such as *S. enteritidis* and *S. typhimurium*, which collectively cause an estimated 93.8 million cases of gastroenteritis and approximately 155,000 deaths annually worldwide (Gal-Mor et al., 2014; Afendy Abdul Talib et al., 2022). The increasing antimicrobial resistance in *Salmonella* is especially alarming, with resistance rates reaching 50–80% for antibiotics such as sulfonamides, ampicillin, and tetracyclines (Mąka et al., 2015). Third-generation cephalosporins and quinolones have served as the primary treatment options for multidrug-resistant (MDR) infections. However, many serotypes have become resistant to them and are thus associated with more severe and fatal infections (Teklemariam et al., 2023). This resistance is largely attributed to the extensive and unnecessary usage of antimicrobial agents for treating infectious diseases in animals and humans, and as promoters of growth in livestock production (Van Boeckel et al., 2019; Huber et al., 2021). This has prompted the World Health Organization to list antimicrobial-resistant *Salmonella* among the top ten high-priority pathogens (Fong et al., 2020).

Given the lack of novel antibiotic development, new therapeutic strategies are needed. Researchers have again shed light on bacteriophages as a promising alternative to antibiotics due to their bactericidal activity, host specificity, self-limiting capabilities, and ease of genetic manipulation (Khan and Rahman, 2022). When applied to *Salmonella*, many studies have demonstrated their capacity to lyse MDR isolates (Lopez-Garcia et al., 2023). The Food and Drug Administration (FDA) has designated certain bacteriophage products as generally recognized as safe (GRAS), allowing their use in livestock and poultry (Mandeville, 2024). Several studies have demonstrated that bacteriophage cocktails can effectively reduce bacterial contamination when applied directly to feed, raw meat, or vegetables. Additionally, phages have been successfully used as therapeutic agents in cases where antibiotic treatment was ineffective (Almashtoub et al., 2025). Moreover, other studies have demonstrated that phages may help restore antibiotic sensitivity in certain isolates, a phenomenon known as phage-antibiotic synergy (Cui et al., 2024). However, to successfully translate phage therapy and its applications, a detailed understanding of phage–host interactions, particularly receptor recognition, is essential, as receptor usage determines host range, shapes resistance evolution, and influences the therapeutic stability of phage treatment (Labrie et al., 2010).

In this study, we describe the isolation and detailed characterization of a newly isolated lytic bacteriophage, GF04, against *Salmonella enterica* serovar Enteritidis. Additionally, after isolating a spontaneous phage-resistant host variant, comparative genomic analysis revealed a mutation in a membrane-associated gene linked to resistance, strongly implicating *yjiK_2* as a candidate determinant of resistance and a plausible receptor-associated factor for GF04. These findings provide insight into receptor-mediated host specificity and resistance evolution, and establish a foundation for future functional validation of the GF04 receptor.

## 2 Materials and methods

### 2.1 Bacterial strains

Nineteen *S. enteritidis* strains clinically isolated in Lebanon were provided by the Bacteriology and Molecular Microbiology Laboratory, Department of Experimental Pathology, Immunology, and Microbiology, American University of Beirut. The *S. enteritidis* strains used in this study are identical to those described previously (Almashtoub et al., 2025); their characteristics, including sequence type, plasmid content, and antimicrobial susceptibility profiles, are summarized in Supplementary Tables A1-A3. Each strain was cultured on *Salmonella-Shigella* (SS) agar, subcultured on Luria–Bertani (LB) agar, and subsequently stored in 50% glycerol at −20 °C.

### 2.2 Isolation and purification of GF04 bacteriophage

Enrichment was carried out by incubating overnight at 37 °C a mixture of 10 mL of the sewage water, 2 mL LB broth, and 2 mL of a 0.5 McFarland bacterial suspension. The enrichment was centrifuged at 4000 x g for 15 min, and the supernatant was filtered through a 0.2 μm syringe filter (Van Twest and Kropinski, 2009).

A conventional plaque assay was performed by carrying out serial tenfold dilutions (1:10) of the filtrate in LB broth, then mixing them with 1 mL of a 0.5 McFarland bacterial suspension and 3 mL of 0.6% top agar maintained at 50 °C, followed by pouring onto LB agar plates and overnight incubation at 37 °C.

A single, well-isolated plaque was picked and transferred into an Eppendorf tube containing 500 μL of SM buffer (100 mM NaCl, 8 mM MgSO₄·7H₂O, and 50 mM Tris–HCl [1 M, pH 7.5]). The Eppendorf was then centrifuged at 8000 x g for 5 min to pellet agar debris. 100 μL of the supernatant was enriched with 10 mL LB Broth and 2 mL of 0.5 McFarland bacterial suspension overnight at 37 °C (Almashtoub et al., 2025). Three successive rounds of picking, enrichment, filtration, and plaque assay were done to obtain a pure phage clone. Purified bacteriophage stocks were stored at 4 °C, and the final stock used for downstream experiments had a titer of approximately 10^10^ PFU/mL, as determined by triplicate plaque assays on plates containing 30–300 plaques using the following formula (Kropinski et al., 2009):

$\mathrm{PFU}/\mathrm{mL}=\frac{\text{Mean Number of plaques}\times \text{Dilution factor}}{\text{Volume plated}\;(\mathrm{mL})}$

### 2.3 Bacteriolytic activity assay

The bacteriolytic activity of GF04 was tested at multiplicities of infection (MOI) = 10, 1, 0.1, and 0.01 in triplicate in a 96-well round-bottom plate (Samanta and Datta, 2025). MOI was calculated as the ratio of the number of infectious viral particles to the number of target cells (Gadagkar and Gopinathan, 1980). Test wells consisted of 100 μL of phage lysate adjusted to the desired concentration to achieve MOIs of 10, 1, 0.1, and 0.01, mixed with 70 μL of a 0.5 McFarland bacterial suspension (~1 × 10^8^ CFU/mL). Positive control (PC) wells contained 70 μL of 0.5 McFarland bacterial suspension with 100 μL of LB broth. Negative control (NC) wells contained either 170 μL of LB broth alone or 170 μL of phage lysate alone. The plate was incubated in a microplate reader at 37 °C for 12 h, with OD₆₀₀ measured every 30 min.

### 2.4 Host range determination by efficiency of plating (EOP)

The host range of GF04 was determined via efficiency of plating (EOP) by spotting 3 μL of 10-fold serially diluted phage lysates onto bacterial lawns. Plaques were counted at appropriate dilutions, and PFU/mL values were calculated. EOP was expressed as the ratio of PFU/mL on each test strain relative to the reference host SAL271 (10^10^ PFU/mL) (Kuźmińska-Bajor et al., 2021). Strains with measurable EOP values (EOP > 0) were considered permissive hosts, indicating productive infection. EOP values were defined as high when EOP ≥ 0.5, moderate when 0.01 ≤ EOP < 0.5, and low when 0.0001 < EOP < 0.01 (Kuźmińska-Bajor et al., 2021).

### 2.5 Adsorption rate assay

The adsorption rate assay experiment was carried out at an (MOI) of 1. Nine hundred μL of 0.5 McFarland bacterial suspension was added to 7 Eppendorf tubes labeled *t* = 30 min, *t* = 25, *t* = 20… *t* = 5, *t* = 3 min. Beginning at the tube labeled *t* = 30 min, 100 μL from the phage stock was sequentially added to each tube at 5-min intervals, proceeding in reverse order until the 3-min tube. A time-zero control contained 100 μL phage and 900 μL LB broth only, serving to determine how many plaques are present initially. Samples were simultaneously centrifuged at 8000 x g for 5 min at 4 °C, the pellet was discarded, and the supernatant was diluted to the desired concentrations. For plaque enumeration, each dilution was mixed with 1 mL of a 0.5 McFarland bacterial suspension and 3 mL of LB top agar (0.6% agar), then poured onto LB agar plates and incubated overnight at 37 °C. The percentage of adsorbed phages at each time point was then determined.

### 2.6 One-step growth curve

A one-step growth experiment was conducted at a multiplicity of infection (MOI) of 1 using an initial bacterial concentration of 1 × 10^8^ cells/mL. Briefly, 9 mL of 0.5 McFarland bacterial suspension was centrifuged for 15 min at 8000 x g and resuspended in LB broth prior to infection with 1 mL of the phage stock. After 5 min, this mixture was centrifuged at 8000 x g for 15 min. After the supernatant was discarded, 10 mL of LB broth was added to the pellet. 500 μL of the bacteria-bacteriophage mixture was placed in an Eppendorf tube, centrifuged at 10,000 x g for 5 min, then the supernatant was discarded, and the pellet was resuspended in 500 μL LB broth. The same process was repeated every 5 min till *t* = 70 min. All the Eppendorfs were serially diluted to the desired concentration. For plaque enumeration to determine PFU titer (PFU/mL), each dilution was mixed with 1 mL of a 0.5 McFarland bacterial suspension and 3 mL of LB top agar (0.6% agar), then poured onto LB agar plates and incubated overnight at 37 °C. The burst size was then calculated according to the following formula (Ellis and Delbrück, 1939):

$\text{Burst size}=\frac{\mathrm{PFU}\;\mathrm{at}\;\text{plateau}-\mathrm{PFU}\;\mathrm{at}\;t=0}{\text{Total number of infected cells}}$

### 2.7 Phenol-chloroform extraction of phage DNA

500 μl of the phage lysate (10^10^ PFU/mL) was treated with 5 μL DNase I and 1 μL RNase A (1/10, 10 mg/mL) and incubated for 1.5 h at 37 °C to remove contaminating nucleic acids. Enzymes were inactivated by adding 20 μL of 0.5 M EDTA to the tube, followed by incubation at 75 °C for 15 min. For capsid digestion, 2 μL of proteinase K (20 mg/mL) was added, then incubated for 1 h at 56 °C. 50 μL of 10% SDS was added, and the mixture was further incubated for one hour at 56 °C. The digested mixture was treated with 580 μL phenol/chloroform/isoamyl alcohol (25:24:1), mixed gently, and centrifuged at 10,000 × g for 10 min at room temperature. The aqueous phase containing the target DNA was then collected. An equal volume of chloroform/isoamyl alcohol (24:1) was added, then centrifuged for 10 min at 10,000 × g, and the aqueous phase was collected. To precipitate the DNA, 1/10 volume of 3 M sodium acetate and 2.5 volumes of cold 100% ethanol were added to the collected aqueous phase, then incubated for 1 h at −20 °C. A final centrifugation for 10 min at 4,668 x g was performed, and the supernatant was discarded. The DNA pellet was washed twice with 70% ethanol, followed by heating at 50 °C to dry, and then resuspended in 20 μL MQ water. The quantification and purity of the obtained phage DNA were measured by Qubit™ Fluorometer using the Qubit™ dsDNA HS Assay Kit (Thermo Fisher Scientific, Waltham, MA, USA) according to the manufacturer’s instructions and by Nanodrop, respectively.

### 2.8 Phage whole genome sequencing and analysis

Whole genome sequencing (WGS) of the isolated bacteriophage was carried out using the Rapid Barcoding Kit 24 V14 (SQK-RBK114.24; Oxford Nanopore Technologies, UK) according to the manufacturer’s protocol. Using the Galaxy European Server https://usegalaxy.eu/user, the quality of the raw FastQ nanopore reads was checked via the Falco tool. The Filtlong tool was used to filter the data. The resulting filtered FASTQ high-quality reads were assembled using the Flye tool. Assembly quality was assessed using QUAST. Genome annotation was performed using Pharokka. The proteomic tree of the bacteriophage was constructed using ViPTree,^1^ which generates viral phylogenetic trees based on whole genome sequence similarities (Nishimura et al., 2017). The PhageLeads server^2^ was used to predict toxins, virulence, or antimicrobial resistance genes (Yukgehnaish et al., 2022). In addition, functional annotation and screening for antimicrobial resistance and virulence-associated genes were further supported using the PhageScope platform (Wang et al., 2024). To assess the presence of lysogeny-associated functions, predicted protein sequences were analyzed using InterProScan to identify conserved domains, with particular attention to integrases, recombinases, excisionases, and transcriptional repressors (Blum et al., 2025; Paysan-Lafosse et al., 2025). To determine the intergenomic similarity between the isolated phage and other closely related *Salmonella*-infecting bacteriophages, the Virus Intergenomic Distance Calculator (VIRIDIC) was used^3^ (Moraru et al., 2020).

### 2.9 Isolation of a phage-resistant population for receptor identification

To identify bacterial genetic determinants associated with phage resistance and infer the candidate phage receptor, a phage-resistant (R) population was isolated directly following phage challenge rather than long-term experimental evolution. The ancestral wild-type (WT) strain was exposed to the lytic phage, and resistant survivors were identified using a spot assay on bacterial lawns. Briefly, WT bacteria were plated to form a confluent lawn and spotted with phage suspensions. After incubation, colonies emerging within or adjacent to cleared lysis zones were picked as candidate resistant colonies, subcultured on fresh LB agar plates, and then tested by plaque assay to confirm the resistant phenotype prior to genomic analysis. The ancestral WT strain was processed in parallel as the reference comparator.

### 2.10 Comparative phage adsorption assays

Adsorption assays were performed on both the wild-type strain (SAL271 WT) and the resistant strain (SAL271R) under identical experimental conditions. Bacterial suspensions (0.5 McFarland, ~1 × 10^8^ CFU/mL) were mixed with phage stock (~10^10^ PFU/mL) at a multiplicity of infection (MOI) of 1. Samples were collected at 0, 5, 10, and 15 min post-incubation. At each time point, aliquots were centrifuged (8,000 × g for 5 min at 4 °C) to pellet bacterial cells, and the supernatants containing unadsorbed phages were collected. Residual phage titers were then determined by plaque assay (PFU/mL). A time-zero control was included to account for the initial phage input before adsorption.

### 2.11 Phenol-chloroform extraction of bacterial DNA

Resistant and wild-type bacterial cultures (1.5 mL) were pelleted at 10,000 x g for 10 min and resuspended in 500 μL nuclease-free water. Then, 2 μL of RNase A was added and incubated for 1 h at 37 °C, followed by the addition of 50 μL of EDTA and incubation for 15 min at 37 °C to inactivate RNase A. 10 μL of proteinase K (20 mg/mL) was added and then incubated for 1 h at 55 °C. This was followed by the addition of 50 μL of 10% SDS for 30 min at 55 °C. The mixture was then treated with 610 μL of phenol/chloroform/isoamyl alcohol (25:24:1), followed by careful mixing (no vortexing) and centrifugation at room temperature for 10 min at 10,000 x g. The aqueous phase was collected, and an equal volume of chloroform/isoamyl alcohol (24:1) was added, followed by careful mixing, centrifugation for 10 min at 10,000 x g, and collection of the aqueous phase. DNA was precipitated with 1/10 volume of 3 M sodium acetate and 2.5 volumes of cold 100% ethanol and incubated for 1 h at −20 °C, then centrifuged at 4,668 x g for 10 min. The DNA pellet was washed twice with 200 μL of 70% ethanol, dried at 50 °C, and resuspended in 20 μL MQ water. The quantification and purity of the obtained bacterial DNA were measured by Qubit™ Fluorometer using the Qubit™ dsDNA HS Assay Kit (Thermo Fisher Scientific, Waltham, MA, USA) according to the manufacturer’s instructions and by Nanodrop, respectively.

### 2.12 Comparative whole genome sequencing of the wild-type and resistant population

Paired-end WGS was performed on an Illumina NovaSeq 6,000 platform, generating 150-bp paired-end reads. Sequencing was conducted to achieve an average genome-wide depth of at least 20 × coverage for both WT and resistant samples. Raw Illumina reads were quality-trimmed and filtered using Trimmomatic v0.40 (Bolger et al., 2014). Quality-filtered reads were aligned to the WT reference genome assembly using BWA-0.7.19 with default settings (Li and Durbin, 2009). Alignment files were converted, sorted, and indexed using SAMtools v1.23 (Li et al., 2009). Single-nucleotide variants (SNVs) and small insertions/deletions (indels) were identified using Breseq v0.39.0 (Deatherage and Barrick, 2014). When analyzing mixed populations, Breseq was run in polymorphism mode; in the present study, variant calling was performed on the resistant population relative to the WT reference. Only variants supported by a minimum read depth of 10 × at the corresponding genomic position were retained. Candidate resistance-associated variants were defined as mutations detected in the resistant population and absent from the WT strain. Variants were annotated using the WT reference annotation (GFF) generated with Prokka v1.15.6 (Seemann, 2014), and classified by genomic context (coding sequence, intergenic region, or RNA feature) and predicted effect (synonymous, non-synonymous, frameshift, etc.). Variant frequencies were calculated as the proportion of reads supporting the alternate (mutant) allele relative to total coverage at each locus. Genomic distribution plots (including Manhattan-style plots of mutation frequency across genomic coordinates) were generated using Python 3.14. Data parsing and analysis were performed with pandas, numpy, and scipy, while visualization was performed with matplotlib and seaborn. Where applicable, statistical summaries and plotting scripts were executed in a reproducible workflow using Jupyter notebooks.

### 2.13 AlphaFold-based structural modeling of the *yjiK*_*2* protein

The three-dimensional structure of the *yjiK*_2 protein was predicted using AlphaFold (Jumper et al., 2021). The amino acid sequence of *yjiK*_2 from *Salmonella enterica* serovar Enteritidis strain SAL271 was used as input for structure prediction. Both the wild-type and mutant sequences containing the P128L substitution were modeled independently to evaluate potential structural effects associated with the mutation. Predicted protein structures were visualized and compared to assess potential conformational differences that could influence receptor accessibility or phage-binding interactions.

### 2.14 Statistical analysis

Adsorption assay data were analyzed by two-way analysis of variance (ANOVA), with bacterial strain (wild-type and resistant) and time (0, 5, 10, and 15 min) as independent variables. Data are presented as mean ± SD of three independent biological replicates. When significant effects were observed, *post hoc* pairwise comparisons between strains at individual time points were performed with Holm-adjusted *p*-values to account for multiple testing. Results are expressed as mean ± SD, and *p* < 0.05 was considered statistically significant.

## 3 Results

### 3.1 Bacteriophage isolation and purification

**Figure 1:**
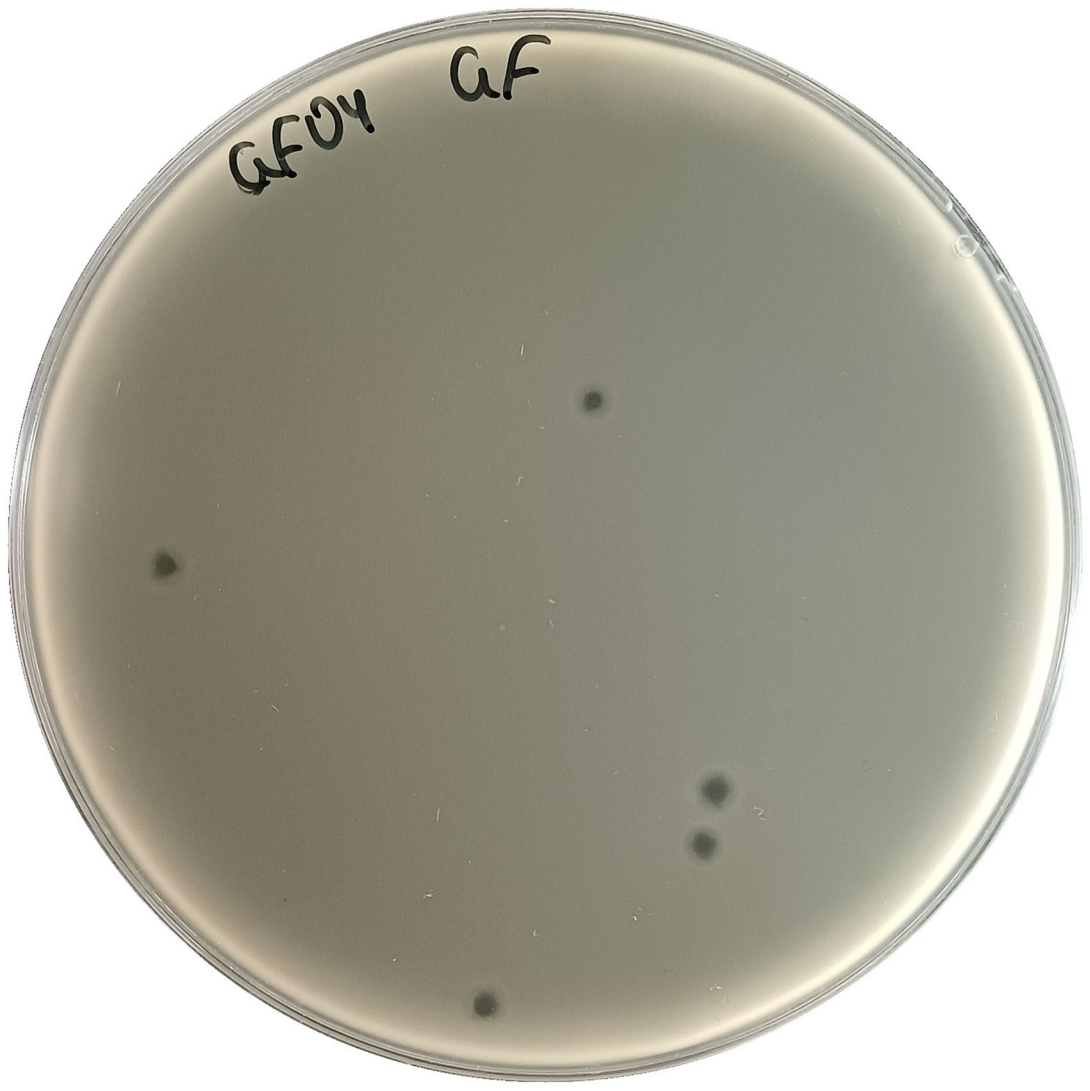
Morphological characteristics of Salmonella phage GF04. Plaques of a diameter of ~3 mm in diameter produced by Salmonella phage GF04 on SAL271 bacterial lawn after three rounds of purification on a double-layer agar plate.

A bacteriophage targeting *Salmonella enterica* serovar Enteritidis strain SAL271 was isolated from sewage samples. Following enrichment with its host, a double-layered agar (plaque) assay revealed clear plaques of ~3 mm in diameter, confirming the presence of bacteriophages. A single plaque was isolated and enriched overnight with its host strain SAL271 three consecutive times to ensure phage purity and genetic uniformity. The purified isolate was subsequently named Salmonella phage GF04 (Figure 1).

### 3.2 Bacteriolytic activity of GF04

**Figure 2:**
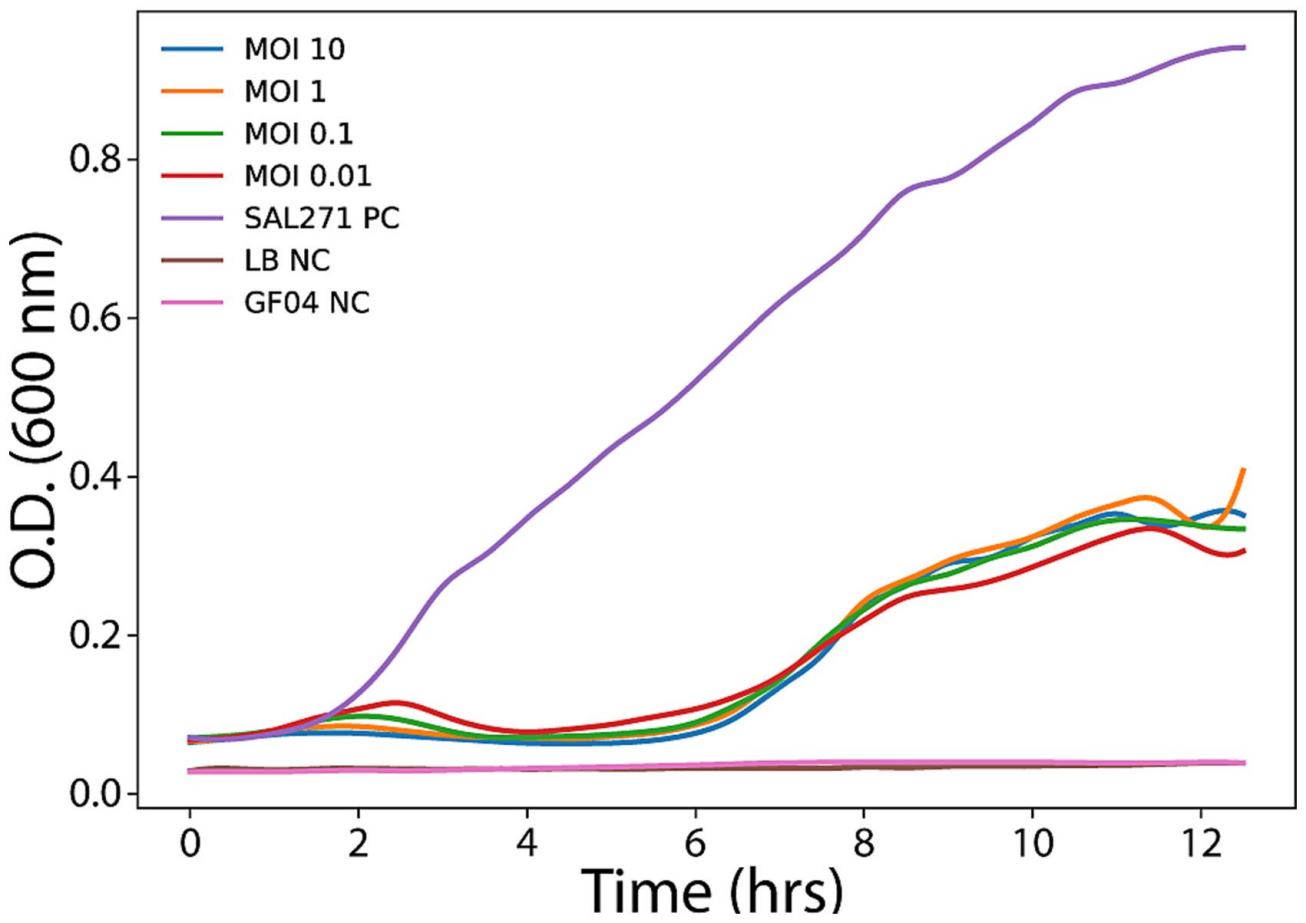
Bacteriolytic activity of the phage at different multiplicities of infection (MOIs). Bacterial growth was monitored by measuring optical density at 600 nm (OD_600_) over 12 h following infection with the phage at the indicated MOIs. An uninfected culture served as the positive control (SAL271 PC). The untreated control exhibited normal exponential growth, reaching a high OD_600_ by the end of the experiment. In contrast, phage-treated cultures showed suppressed bacterial growth in an MOI-independent manner. All MOIs resulted in rapid inhibition of bacterial proliferation, reflected by reduced OD_600_ values compared to the positive control. Partial regrowth observed at later time points in some conditions may indicate the emergence of resistant subpopulations. These results demonstrate the dose-independent bacteriolytic activity of GF04. NC: Negative control.

The untreated control culture exhibited typical growth kinetics, entering the exponential phase after approximately 3–4 h and reaching a high optical density by the end of the experiment. In contrast, all phage-treated cultures showed marked suppression of bacterial growth compared to the control. Notably, the growth inhibition profiles were highly similar across the different MOIs tested, indicating that bacteriolytic activity was largely MOI-independent under the experimental conditions. Infected cultures displayed reduced exponential growth and lower final OD₆₀₀ values relative to the untreated control, with only limited bacterial regrowth observed at later time points, which may be consistent with the emergence of phage-resistant variants of SAL271 (Figure 2). These findings suggest that the phage exerts robust lytic activity across a range of infection doses, consistent with efficient replication dynamics and effective bacterial killing once infection is established.

### 3.3 Host range of GF04 by efficiency of plating (EOP)

**Figure 3:**
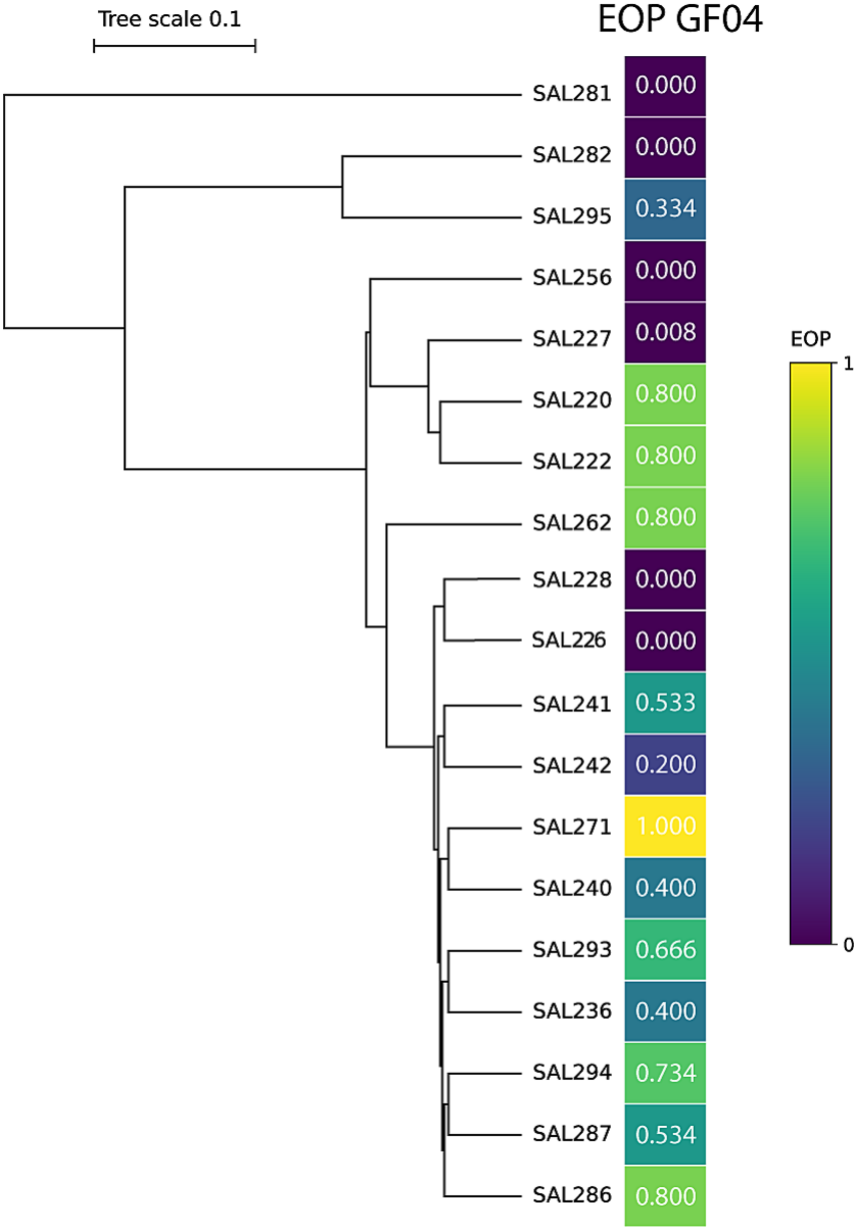
Phylogenetic relationship of *Salmonella* isolates and efficiency of plating (EOP) profile of Salmonella phage GF04 using SAL271 as the reference host. A phylogenetic tree of the tested *Salmonella* isolates is shown with a corresponding heatmap indicating the efficiency of plating (EOP) of GF04 phage for each of the 19 strains. EOP values range from 0 to 1, where 1 indicates plating efficiency comparable to the reference host (SAL271), and 0 indicates no detectable plaque formation. The color scale represents increasing EOP from dark purple (low/no EOP) to yellow (high EOP).

Efficiency of plating (EOP) analysis revealed that phage GF04 productively infected 14 out of 19 tested *Salmonella* isolates, indicating a relatively broad but variable host range within the studied collection (Figure 3). Susceptible strains having an EOP value greater than 0 were distributed across distinct branches of the dendrogram rather than confined to a single clade, suggesting that phage infectivity is not strictly lineage-restricted. Conversely, several closely related isolates remained resistant, highlighting variability in susceptibility even among genetically related strains. EOP values varied substantially across isolates, ranging from no detectable plaque formation (EOP = 0) to high efficiency (up to 0.8 relative to the reference host SAL271). Several isolates, including SAL220, SAL222, SAL262, and SAL286, showed high susceptibility (EOP = 0.8), whereas others showed moderate to low EOP values, indicating reduced infection efficiency. In contrast, strains such as SAL281, SAL282, SAL256, SAL228, and SAL226 showed no detectable EOP, suggesting an absence of productive infection. Together, these findings indicate that while GF04 exhibits cross-strain infectivity, host specificity is likely influenced by strain-level factors, such as receptor availability or surface structure variability, rather than overall phylogenetic proximity.

### 3.4 Infection kinetics of GF04

**Figure 4:**
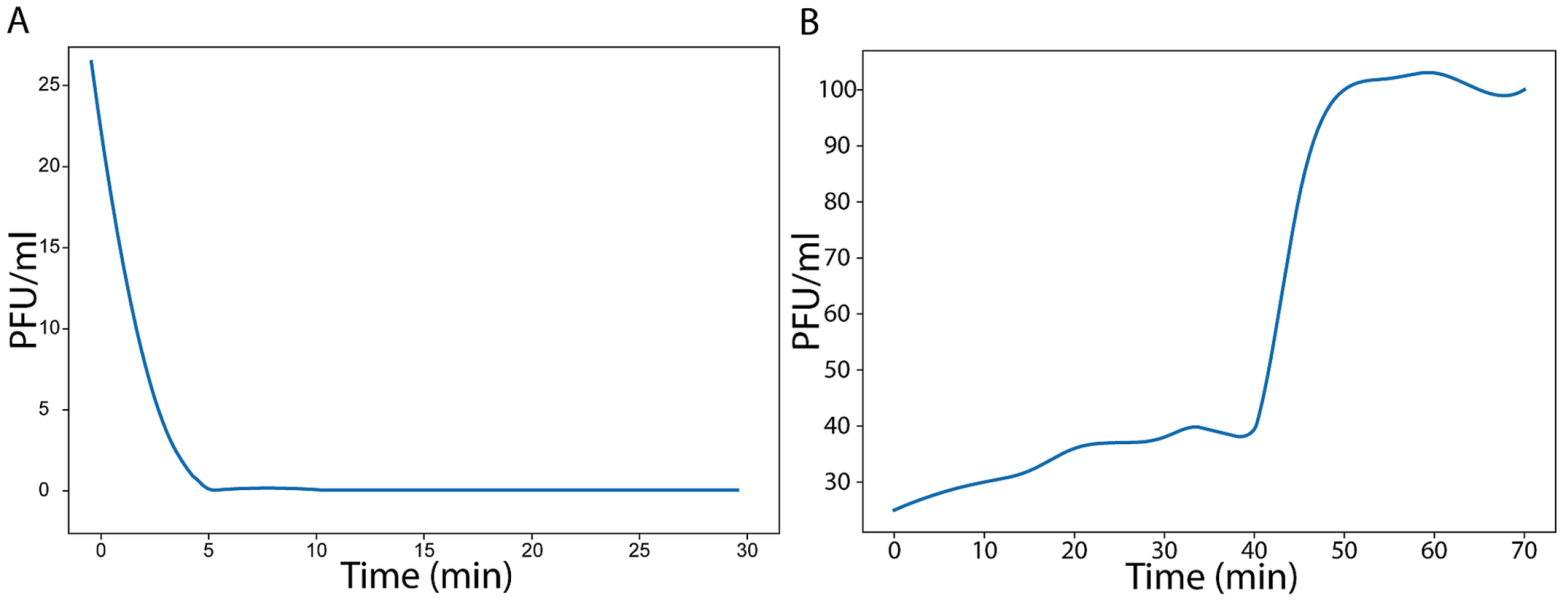
Bacteriophage-host interaction dynamics. **(A)** Phage adsorption was evaluated by incubating phage particles with exponentially growing bacterial cells and quantifying the number of free (non-adsorbed) phages remaining in the supernatant at the indicated time points. Free phage titers (PFU/mL) were determined using the double-layer agar plaque assay. A rapid decrease in PFU/mL was observed within the first minutes of incubation, indicating efficient phage attachment to the bacterial surface. The plateau reached after approximately 5 min suggests near-complete adsorption under the experimental conditions. **(B)** One-step growth curve performed at MOI = 1 with an initial bacterial density of 1 × 10^8^ cells/mL. Following synchronized infection and removal of unadsorbed phages, extracellular titers (PFU/mL) were determined by double-layer agar assay. A latent period of ~45 min was observed, followed by a rise phase beginning at ~45 min. Titers reached a plateau at 55–60 min, indicating completion of the first lytic cycle. The increase in phage concentration from 35 × 10^8^ PFU/mL at t₀ to 100 × 10^8^ PFU/mL at the plateau corresponds to an estimated burst size of approximately ~103 PFU per infected cell under the experimental conditions.

As shown in Figures 4A, a rapid decline in free phage particles was observed during the first minutes of incubation. The PFU/mL decreased sharply within the initial 3–5 min, indicating efficient phage attachment to the bacterial surface. By approximately 5 min, the majority of phage particles had adsorbed to host cells, with PFU values approaching near-zero levels. After this initial rapid phase, the curve reached a plateau, suggesting that maximal adsorption had been achieved and that only a negligible fraction of free phage remained in suspension. The steep slope observed at early time points reflects a high adsorption efficiency and a strong affinity of the phage for its bacterial receptor. Overall, these results demonstrate that the phage exhibits fast and efficient adsorption kinetics, with most viral particles attaching to host cells within the first few minutes of contact.

Additionally, the one-step growth experiment revealed thatphage counts remained stable during the initial phase of infection, defining a latent period of ~45 min, during which phage genome replication, protein synthesis, and virion assembly occurred intracellularly without detectable release of progeny particles into the medium. A pronounced increase in extracellular PFU/mL was observed beginning at ~45 min, marking the onset of the rise phase. Phage titers rapidly increased and reached a plateau by approximately ~55–60 min, indicating completion of the first lytic cycle. The phage concentration increased from 35 × 10^8^ PFU/mL at the start of the experiment (t₀) to 100 × 10^8^ PFU/mL at the plateau, corresponding to a net production of 6.5 × 10^9^ PFU/mL (Figure 4B). Considering the initial infection conditions, this increase translates into an estimated burst size of approximately ~103 plaque-forming units per infected cell, demonstrating robust intracellular replication and efficient release of progeny virions.

### 3.5 Genomic organization and phylogenetic placement of Salmonella phage GF04

**Figure 5:**
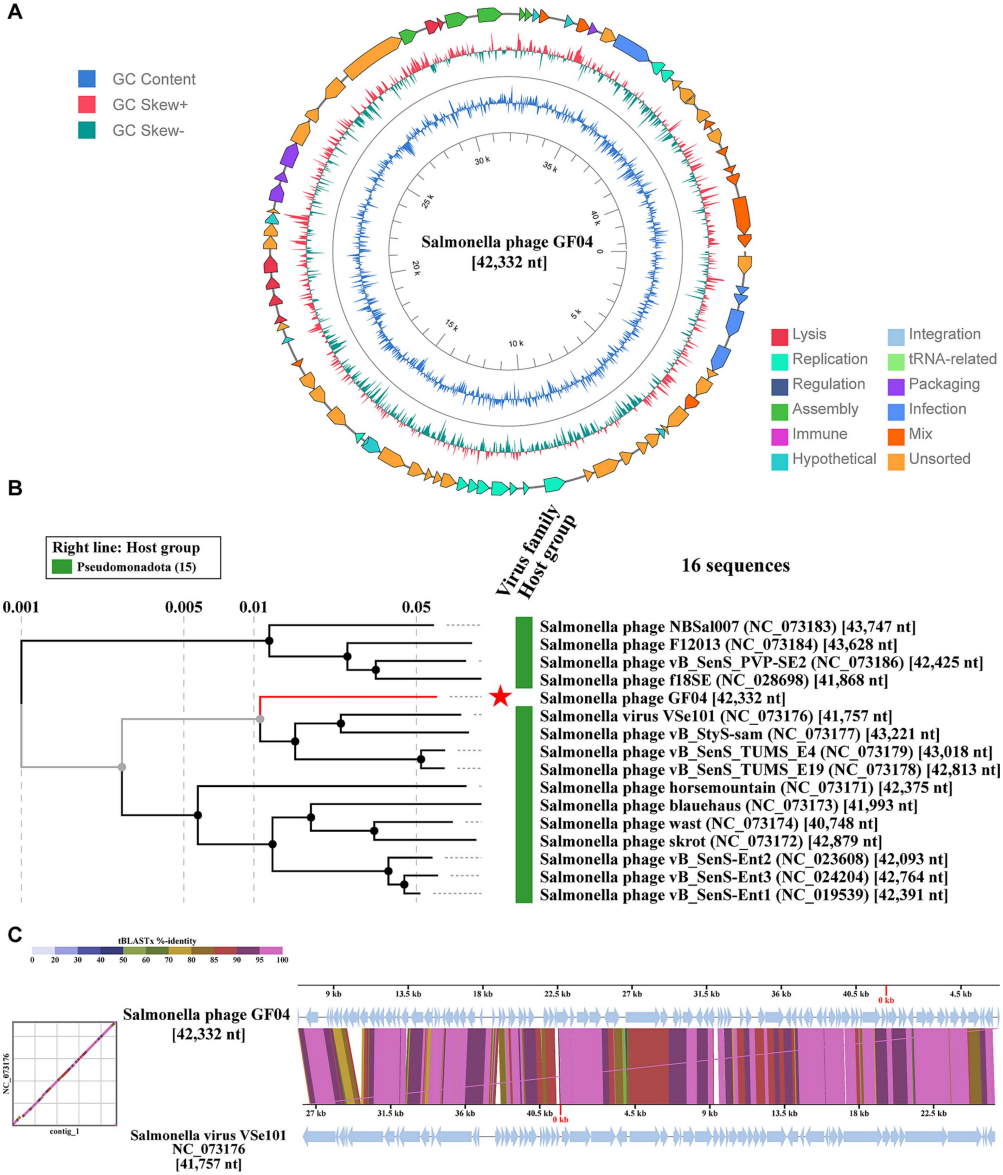
Genomic and phylogenetic characterization of Salmonella phage GF04. **(A)** Circular genome map of GF04 (42,332 bp) showing annotated open reading frames color-coded by functional category. Inner rings represent GC content and GC skew distribution across the genome. **(B)** Whole-genome phylogenetic tree of GF04 and 15 related phages (16 total sequences), illustrating clustering within a *Salmonella*-infecting phage lineage. Scale bar represents nucleotide substitutions per site. **(C)** Whole-genome tBLASTx comparison of GF04 with closely related phage, demonstrating conserved synteny and high percentage identity across the genome. Color gradients correspond to sequence similarity.

The complete genome of Salmonella phage GF04 is 42,332 bp in length and exhibits a modular organization typical of lytic *Salmonella*-infecting phages. Figure 5A shows the circular genome map of GF04 with open reading frames annotated and color-coded according to predicted functional categories, including replication, structural assembly, DNA packaging, regulation, host lysis, and hypothetical proteins. Functional modules are arranged in a conserved order, with structural and packaging genes clustered together and replication-associated genes localized within a distinct genomic region. GC content and GC skew analyses display a relatively uniform compositional pattern across the genome, without major discontinuities suggestive of large-scale horizontal gene acquisition. Genomic screening using PhageLeads and PhageScope servers demonstrated that GF04 does not possess any detectable genes related to virulence, antimicrobial resistance, or lysogeny, further supporting the putative lytic nature of GF04. These findings were consistent with InterProScan and Pharokka analysis, which did not reveal any conserved protein domains or functional annotations associated with lysogeny-related functions, including integrases, recombinases, excisionases, or CI-like transcriptional repressors (Supplementary Table S1).

The complete GF04 phage sequence has been submitted to GenBank with a Gb ref. PX241559.

Figure 5B presents the whole genome phylogenetic tree constructed from 15 related phage genomes. GF04 clusters within a well-supported clade comprising *Salmonella-inf*ecting phages, including *Salmonella vir*us VSe101 and vB_SenS-Ent1/2/3, among others. Branch lengths indicate close evolutionary relatedness, and the grouping reflects strong genomic similarity within this lineage. All members of this cluster share comparable genome sizes (~41.7–43.7 kb), consistent with the size of GF04. Based on this alignment, it is concluded that GF04 belongs to the genus *Jerseyvirus*, subfamily *Guernseyvirinae*, family *Sarkviridae*, and class *Caudoviricetes*.

Figure 5C illustrates the whole genome tBLASTx comparison between GF04 and closely related phages. Extensive sequence conservation and synteny are observed across most of the genome, with high identity values throughout structural and replication modules. Divergence is limited to discrete genomic regions, likely corresponding to accessory or host-interaction genes. The strong conservation pattern further supports the phylogenetic placement of GF04 within this defined *Salmonella pha*ge lineage. To further evaluate its taxonomic position, the genome of Salmonella phage GF04 was compared with representative genomes from the genus *Jerseyvirus* obtained in the phylogenetic tree using the VIRIDIC tool (Supplementary Figure S1). Pairwise nucleotide identities ranged from 83.6 to 90.4%. These values are below the 95% species-level cutoff but exceed the 70% genus-level threshold, suggesting that GF04 likely represents a distinct species within the genus *Jerseyvirus, fa*mily *Sarkviridae. Co*llectively, genomic organization, phylogenetic clustering, and whole genome similarity analyses confirm that GF04 belongs to a closely related group of lytic *Salmonella-inf*ecting phages and shares extensive structural and evolutionary conservation with previously described members of this lineage.

### 3.6 Isolation of a GF04-resistant *Salmonella* derivative

Following exposure of SAL271 to phage GF04, bacterial colonies emerged within lysis zones on the bacterial lawn, indicating the presence of spontaneous phage-resistant variants. Subsequent plaque assays confirmed the resistant phenotype of the variant “SAL271R,” as no plaque formation was observed upon re-exposure to GF04 compared with the WT strain, which remained susceptible.

### 3.7 Resistance in SAL271R is associated with impaired phage adsorption

**Figure 6:**
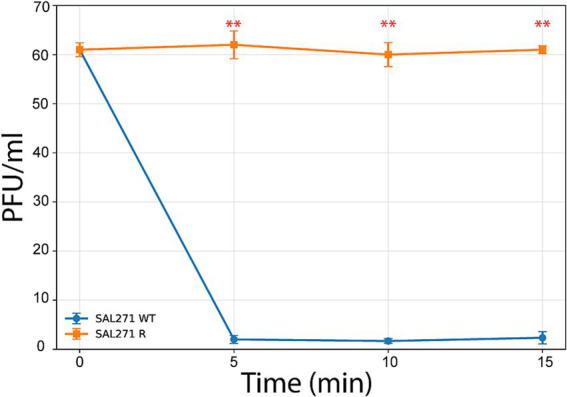
Adsorption efficiency of the phage to wild-type and evolved resistant bacteria. Adsorption assay of phage on SAL271 WT and SAL271 R. Residual phage counts were measured after incubation with SAL271 WT or SAL271 R at 0, 5, 10, and 15 min. Data are shown as mean ± SD of three biological replicates. Phage adsorption decreased rapidly on SAL271 WT, with residual counts dropping markedly by 5 min and remaining low thereafter, whereas residual counts remained essentially unchanged on SAL271 R, indicating impaired adsorption to the mutant strain. Statistical analysis was performed using two-way ANOVA with strain and time as factors, followed by multiple comparisons between strains at each time point with Holm correction. No significant difference was observed at 0 min, whereas significant differences were detected at 5, 10, and 15 min.

Phage adsorption to SAL271 WT occurred rapidly, with residual phage counts dropping to near-zero levels within 5 min and remaining low at subsequent time points. In contrast, residual phage counts in SAL271 R remained largely unchanged over time, indicating a marked impairment in phage adsorption in the resistant strain (Figure 6).

Residual phage counts were analyzed by two-way ANOVA with strain and time as factors. A significant effect of strain was observed [*F*(1,16) = 2968.01, *p* = 1.34 × 10^−19^], along with a significant effect of time [*F*(3,16) = 330.14, *p* = 1.35 × 10^−14^] and a significant strain × time interaction [*F*(3,16) = 329.98, *p* = 1.36 × 10^−14^]. *Post hoc* comparisons with Holm correction showed no difference between strains at 0 min (*p* = 1.000), whereas SAL271 WT differed significantly from SAL271 R at 5 min (adjusted *p* = 0.0015), 10 min (adjusted *p* = 0.0015), and 15 min (adjusted *p* = 1.29 × 10^−^5).

### 3.8 Genome mapping of phage resistance reveals fixation of a missense mutation in *yjiK_2*

**Figure 7:**
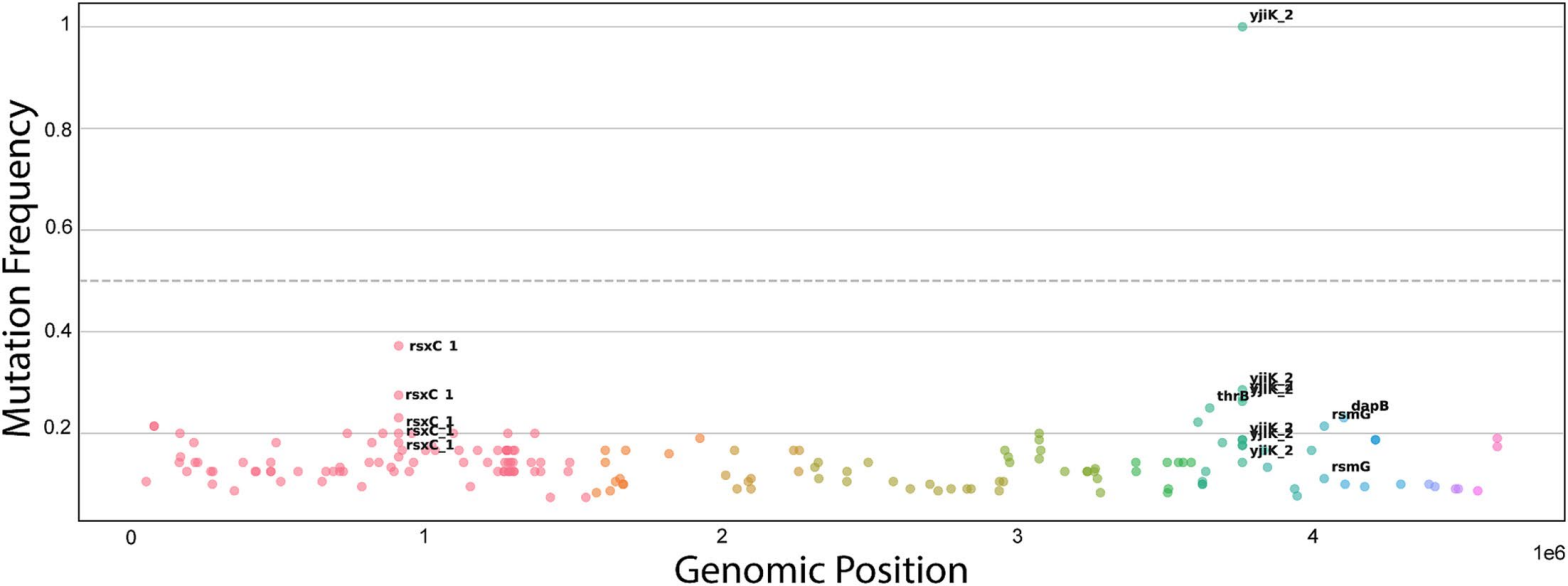
Genomic landscape of phage resistance in the evolved population. Manhattan plot displaying mutation frequency across the bacterial genome. Each point represents a variant, plotted according to genomic position (*x*-axis) and allele frequency (*y*-axis). The fixed mutation in *yjiK*_2_ appears as a prominent spike reaching 100% frequency. Other variants remain at low frequencies across the genome.

To elucidate the molecular determinants of phage resistance and identify the candidate phage receptor, we performed comparative genomic analysis between the ancestral wild-type strain and the phage-resistant evolved population. The mutational landscape was overwhelmingly dominated by a single-nucleotide polymorphism in the *yjiK_2* gene, which reached 100% penetrance (fixation) in the resistant population (Figure 7). This specific mutation consists of a C → T transition that results in a non-synonymous amino acid substitution at residue 128, replacing a rigid, structure-inducing Proline with a flexible, hydrophobic Leucine (P128L). Given Proline’s unique role in constraining peptide backbone conformation and introducing kinks into structural loops, this P128L substitution is predicted to induce a localized structural rearrangement in the *yjiK_2* extracellular or transmembrane domains, potentially influencing receptor binding or accessibility. Corroborating its role as the primary target of phage-mediated selective pressure, *yjiK_2* also exhibited the highest overall mutational burden across the genome, accumulating nine distinct mutation events. While minor secondary variants were detected in other surface-associated elements such as rsxC-1 (~40% frequency), their failure to reach fixation suggests they represent transient, compensatory adaptations rather than the primary mechanism of evasion. Taken together, these data support theleading P128L mutation in *yjiK_2* as the strongest candidate resistance-associated change, and are consistent with the adsorption defect observed in the resistant derivative, supporting a potential role for *yjiK_2* in phage binding or receptor accessibility. However, functional validation will be required to establish whether *yjiK_2* directly serves as the primary receptor for GF04.

### 3.9 Structural modeling of the *yjiK*_2 P128L variant

**Figure 8:**
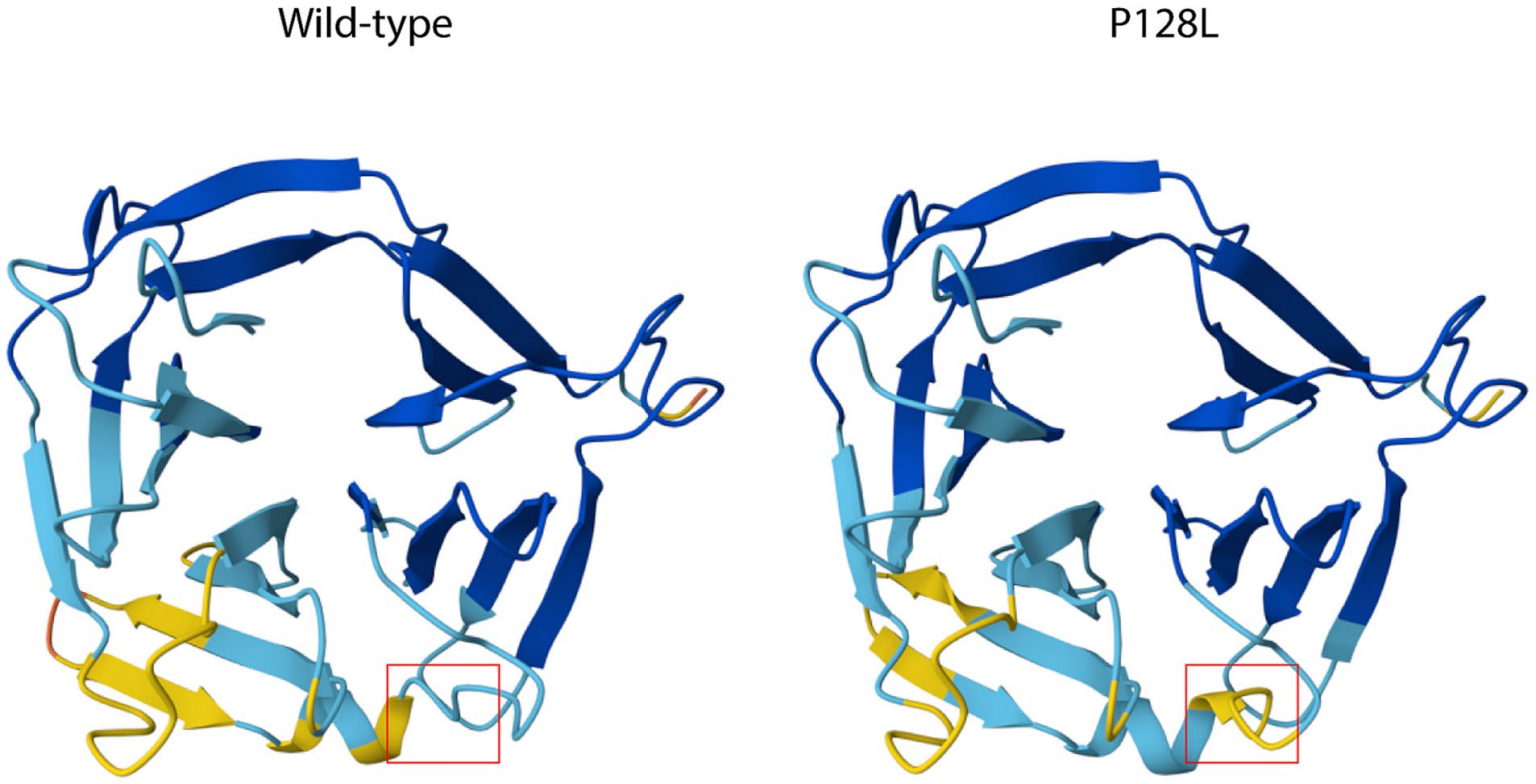
Structural comparison of the *yjiK*_*2* protein between the wild-type and mutant variant. Predicted three-dimensional structures of the *yjiK*_*2* protein generated using AlphaFold are shown for the wild-type (left) and the P12L mutant (right). The mutation site is highlighted with a red box. The substitution Pro128 → Leu induces a local structural rearrangement in the C-terminal loop region, altering the conformation of the surrounding secondary structure elements. Differences are visible in the orientation and flexibility of the loop adjacent to the mutation site, suggesting that the substitution may influence the stability or interaction properties of this region.

Structural comparison between the wild-type and mutant variants revealed that the P128L substitution is located within a membrane-associated region of the protein and is associated with a predicted localized conformational change (Figure 8). In the wild-type model, residue P128 forms part of a structured segment that contributes to the local folding of the protein. Replacement of this rigid proline residue with leucine resulted in a detectable rearrangement of the surrounding residues, altering the orientation of the adjacent structural elements. This predicted alteration could influence local surface topology or receptor accessibility; however, these observations are computational and should be interpreted as hypothesis-generating rather than definitive structural evidence.

## 4 Discussion

In this study, we characterized a lytic *Salmonella*-targeting bacteriophage, GF04, with favorable infection kinetics and a preliminary genomic safety profile, supporting its potential as a therapeutic candidate. Importantly, comparative genomic analysis of a GF04-resistant derivative identified a mutation in the *yjiK_2* gene that is strongly associated with loss of phage adsorption, suggesting a potential role for this gene as a candidate receptor involved in GF04 host recognition.

The isolation of GF04, targeting *S. enteritidis* strain SAL271, from wastewater is consistent with previous studies that have demonstrated the prevalence of *Salmonella* and its phages in urban wastewater systems (Amarillas et al., 2024). In addition, the formation of clear plaques, as seen in Figure 1, is often associated with a strong lytic activity against bacteria, whereas temperate phages usually produce turbid plaques (Hatfull et al., 2022; Amarillas et al., 2024). GF04 displayed similar bacteriolytic profiles across the tested MOIs under the experimental conditions used (Figure 2), attributable to its rapid infection cycle, with complete adsorption within 5 min, a latent period of ~ 45 min, and a burst size of ~103 phages per cell, exceeding that of several reported Salmonella phages (Figure 4) (Amarillas et al., 2024). This means that even if the initial phage concentration is low, the phage population increases significantly after one replication cycle, allowing efficient lysis of the bacterial cells regardless of the starting MOI. The host range analysis based on EOP showed that phage GF04 productively infected 14 of 19 *S. enteritidis* isolates, indicating a relatively broad host range within the tested collection, although validation across a broader and more diverse strain panel is required (Figure 3). Susceptible strains were distributed across multiple phylogenetic branches rather than a single clade, suggesting that infectivity is not strictly determined by bacterial lineage but is more likely influenced by strain-specific surface features such as receptor availability or outer membrane variability. EOP values varied widely, from no detectable infection to high efficiency (up to 0.8 relative to the reference host), highlighting substantial differences in productive infection among hosts. Notably, closely related isolates displayed contrasting phenotypes, with both high susceptibility and complete resistance, further supporting the role of fine-scale strain-level determinants in phage infectivity rather than overall genetic relatedness.

The tBLASTx whole genome alignment demonstrated that GF04 shares several conserved genomic regions with reference members of *Jerseyvirus* (family *Sarkviridae*) (Figure 5C), a group of strictly lytic Salmonella phages commonly isolated from sewage and approved as safe anti-*Salmonella* agents in avian water and feed (Kuźmińska-Bajor et al., 2021). Genome analysis using Pharokka and PhageLeads, complemented by InterProScan, did not identify lysogeny-, virulence-, or antimicrobial resistance-associated genes or conserved protein domains in GF04. These findings further support a preliminary genomic safety profile and are consistent with a putative lytic lifestyle. (Koskella and Brockhurst, 2014). Its lytic nature highlights GF04’s capacity as a promising therapeutic candidate, as temperate phages are generally undesirable in therapy since they may allow bacterial survival through lysogen formation (Hatfull et al., 2022).

Despite these favorable properties, the resistance mechanisms that bacteria employ against phages complicate phage therapy (Li et al., 2022). While post-adsorption defenses such as restriction–modification systems and CRISPR–Cas are common, resistance frequently arises through prevention of adsorption via receptor modification, such as mutation, alteration, or downregulation (Koskella and Brockhurst, 2014; Li et al., 2022). In fact, receptor alteration often emerges rapidly under phage selection, reflecting strong directional pressure on genes encoding phage attachment sites (Venturini et al., 2022).

Several studies have highlighted the importance of phage receptor identification, especially in the process of expanding the therapeutic potential of environmentally sourced phages (Kortright et al., 2020; Mutalik et al., 2020; Adler et al., 2021). Recent advances in phage–host interaction studies have employed high-throughput functional genomics approaches, including transposon sequencing (Tn-seq) (Kortright et al., 2020), random mutant library screening (Shin et al., 2012), and barcoded fitness profiling (RB-TnSeq) (Mutalik et al., 2020), to identify bacterial genes involved in phage infection, including receptor determinants. Determining phage receptors can confirm whether or not a phage can create beneficial trade-offs. A well-characterized example is Pseudomonas phage OMKO1, which targets the OprM component of multidrug efflux pumps in *Pseudomonas aeruginosa* (Chan et al., 2016). Mutations conferring resistance to OMKO1 disrupt OprM function, thereby restoring antibiotic susceptibility. This evolutionary trade-off enhances the therapeutic value of the phage (Strathdee et al., 2023). In *Salmonella*, outer membrane components such as lipopolysaccharide (LPS) and O-antigen are essential for virulence, motility, and antibiotic resistance, while also serving as common receptors for *Salmonella*-targeting phages. Receptor modification to escape phage infection, therefore, often comes at the cost of reduced virulence and increased antibiotic sensitivity (Kong et al., 2011). Furthermore, bacteria developing mutations in a receptor commonly shared by many phages may result in what is known as cross-resistance. Determining these receptors, therefore, provides critical insight into host–phage interactions and helps in designing effective phage cocktails that target distinct receptors, reducing the likelihood of cross-resistance and improving therapeutic success (Adler et al., 2021).

In this context, to identify the candidate receptor of GF04, a GF04-resistant variant was isolated directly from GF04 plaques on the wild-type bacterial lawn. Since these variants originate from the parental host strain itself, they share a common genetic backbone. Thus, any changes conferring resistance are expected to be limited and directly linked to the phage–host interaction. This variant remained resistant in the absence of GF04 selective pressure and exhibited complete loss of adsorption (Figure 6), indicating that resistance likely resulted from structural alteration or functional inactivation of a surface receptor required for phage adsorption. Together, these findings provide evidence that receptor-mediated resistance plays a central role in the GF04–*Salmonella* interaction. Comparative WGS was performed between the ancestral wild-type strain and the phage-resistant population. The alignment process revealed several mutations, dominated by a single-nucleotide polymorphism in the *yjiK*_2 gene, which reached complete fixation (100% allele frequency) in the resistant population (Figure 7). The fixation of this variant identifies it as the leading candidate resistance-associated mutation in the resistant derivative.

The identified mutation is a C → T transition that results in a non-synonymous substitution at residue 128 (P128L). This substitution replaces a proline, a rigid residue that imposes structural constraints on polypeptide backbones, with leucine, a more flexible and hydrophobic residue (Creighton, 1993). This replacement may induce local conformational rearrangements within extracellular or membrane-associated domains of *yjiK_2*, thereby altering receptor-accessibility or spatial orientation.

To investigate whether this substitution could indeed produce structural alterations in the protein, structural modeling of the *yjiK_2* protein was performed using AlphaFold (Jumper et al., 2021). A comparative analysis of the predicted wild-type and mutant structures revealed a localized conformational change in the region surrounding residue 128. Substitution with leucine resulted in a detectable rearrangement of the adjacent loop region, altering the spatial orientation of nearby structural elements. The mutation is predicted to cause a localized structural perturbation that may affect the accessibility or presentation of extracellular domains involved in phage recognition (Figure 8). These structural observations therefore are consistent with the hypothesis that the *yjiK*_2 mutation may contribute to the loss of phage adsorption observed in the resistant strain (Figure 6).

Notably, the *yjiK_2* gene has been annotated across multiple Gram-negative bacteria as a putative membrane protein with no experimentally validated function prior to this study.^4^ There are no studies that have directly linked *yjiK_2* to phage resistance or host receptor function. Therefore, the identification of a fixed *yjiK_2* variant as the dominant mutation in the GF04-resistant population suggests that *yjiK_2* could be involved in phage receptor function. Additionally, the identification of nine distinct mutations in the *yjiK_2* gene further supports strong selective pressure at this locus. Repeated mutational targeting of the same gene under selective pressure is usually a hallmark of adaptive evolution (Lee and Coop, 2017). This means that this gene is under positive selection, and disrupting it confers a fitness advantage under phage exposure. In contrast, additional mutations detected in other surface-associated genes remained at intermediate frequencies and did not reach fixation. Because these variants were not consistently selected across the resistant population, they are unlikely to represent primary resistance determinants. Instead, they may reflect compensatory changes or transient adaptations that occurred during phage exposure. Certain phages utilize secondary receptors for their irreversible attachment to the bacterial host following their initial reversible attachment to their primary receptors. Binding to the secondary receptors primes phages for the release of their DNA (Venturini et al., 2022). Given that secondary receptor interactions typically require specific and essential engagement to permit irreversible infection, the absence of fixation in these loci argues against a central role in phage recognition. Nevertheless, functional validation will be required to determine whether these genes contribute indirectly to adsorption efficiency or post-attachment infection dynamics.

Functional validation is required to definitively confirm whether *yjiK-2* gene serves as the primary receptor utilized by GF04. One method is through site-directed mutagenesis of *yjiK_2* gene in the wild-type strain, followed by phage infection and an adsorption assay, which can confirm whether disrupting this gene alone is sufficient to confer resistance. A clean gene knockout (*ΔyjiK_2*) would provide a complementary approach to confirm the loss-of-function effect. Additionally, providing the resistant strain with a plasmid harboring the wild-type unmutated gene, resulting in the expression of the wild-type receptor, should restore the sensitivity of this strain. Future work should also focus on evaluating the potential fitness trade-offs associated with the emergence of phage resistance in the GF04-resistant variant. In particular, it will be important to assess whether the genetic alteration identified in *yjiK-2*, the potential receptor of GF04, is accompanied by changes in bacterial fitness, virulence, or antibiotic susceptibility. Investigating these aspects would provide valuable insight into the evolutionary and therapeutic implications of resistance to GF04.

This study has some limitations. First, receptor assignment is currently based on comparative genomics, adsorption phenotyping, and structural prediction rather than direct functional validation. Second, host-range testing via EOP was performed with a relatively small and genetically limited panel of *Salmonella enteritidis* isolates, which may limit generalizability across other serovars. Third, genomic safety assessment and structural interpretations are based on in silico predictions and require experimental validation. These aspects represent important directions for future work and will be essential to further evaluate the biological and therapeutic relevance of GF04.

## 5 Conclusion

In summary, GF04 is a newly characterized lytic Salmonella phage with favorable infection kinetics, detectable activity across multiple *S. enteritidis* isolates, and a preliminary genomic safety profile. Comparative analysis of a resistant derivative identified *yjiK*_2 as the leading candidate gene associated with loss of adsorption and phage resistance. Although direct receptor assignment requires functional validation, these findings provide a useful framework for understanding GF04 host recognition and for guiding future phage cocktail design and therapeutic development.

## Data Availability

The raw sequencing data for Salmonella phage GF04 and the associated bacterial isolates have been deposited in publicly accessible databases. The genomic sequence of Salmonella phage GF04 is available in the NCBI GenBank, with an accession number of PX241559. The accession numbers of the *Salmonella enterica* isolates used in this study are provided in Supplementary Table A1.

## Footnotes

1
https://www.genome.jp/viptree/

2
https://phageleads.dk/

3
https://rhea.icbm.uni-oldenburg.de/viridic/

4
https://www.uniprot.org/uniprotkb?query=yjik_2
