# Supplementary material for: Characterization of a lytic Salmonella phage GF04 within the Jerseyvirus lineage and identification of yjiK_2 as a candidate phage receptor
Source: Front Microbiol. 2026 May 14;17:1828982. doi: 10.3389/fmicb.2026.1828982 (PMC13216507; doi:10.3389/fmicb.2026.1828982)
Supplement: Supplementary file 1 [file Supplementary_file_1.docx]

Supplementary Material

# Supplementary Figure


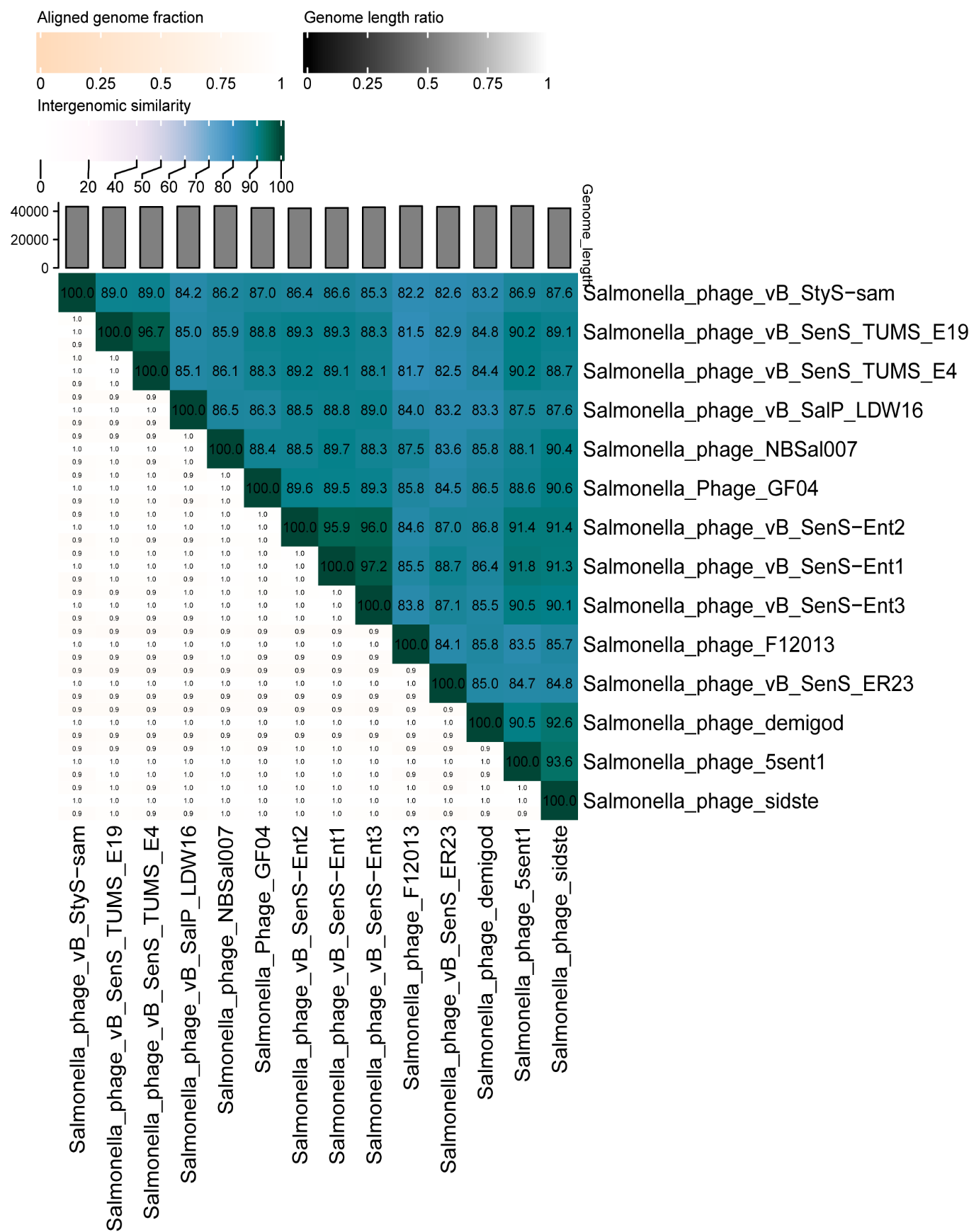


**Supplementary Figure 1. Intergenomic similarity analysis of Salmonella phage GF04 with related *Salmonella* phages using VIRIDIC.** Pairwise intergenomic similarities between GF04 and representative *Salmonella* bacteriophages were calculated using the VIRIDIC (Virus Intergenomic Distance Calculator) tool. The heatmap displays the percentage of nucleotide similarity between phage genomes, with darker colors indicating higher similarity values. The numbers within each cell represent the calculated percentage of genome-wide nucleotide identity. The upper bars represent the aligned genome fraction and genome length ratio between each pair of phages. GF04 shows intergenomic similarity values ranging approximately from 83.6% to 90.4% with the closest related phages. These values fall below the 95% nucleotide identity threshold used for bacteriophage species demarcation, indicating that GF04 represents a distinct viral species within the genus *Jerseyvirus*.

# Supplementary Tables

**Appendix A**

*Appendix A.1*

**Supplementary Table A1. Overview of the *Salmonella enterica* isolates used in this study, including serovar, sequence type (ST), BioProject and BioSample accession numbers, Source, and Plasmid Content.**

| ID | Genus | Serovar | ST | BioProject | BioSample | Source | Plasmids |
| --- | --- | --- | --- | --- | --- | --- | --- |
| SAL220 | Salmonella | Enteritidis | 11 | PRJNA613441 | SAMN43315390 | Human Stool | IncFIB(S) |
| SAL222 | Salmonella | Enteritidis | 11 | PRJNA613441 | SAMN43315391 | Human Stool | IncFIB(S) |
| SAL226 | *Salmonella* | Enteritidis | 11 | PRJNA613441 | SAMN43315395 | Human Stool | IncFIB(S), IncFII(S) |
| SAL227 | Salmonella | Enteritidis | 11 | PRJNA613441 | SAMN43315396 | Human Stool | IncFIB(S) |
| SAL228 | Salmonella | Enteritidis | 11 | PRJNA613441 | SAMN43315397 | Human Stool | IncFIB(S), IncFII(S) |
| SAL236 | Salmonella | Enteritidis | 11 | PRJNA613441 | SAMN43315404 | Human Stool | IncFIB(S), IncFII(S) |
| SAL240 | Salmonella | Enteritidis | 11 | PRJNA613441 | SAMN43315408 | Human Stool | IncFIB(S), IncFII(S) |
| SAL241 | Salmonella | Enteritidis | 11 | PRJNA613441 | SAMN43315409 | Human Stool | IncFIB(S), IncFII(S) |
| SAL242 | Salmonella | Enteritidis | 11 | PRJNA613441 | SAMN43315410 | Human Stool | IncFIB(S), IncFII(S) |
| SAL256 | Salmonella | Enteritidis | 11 | PRJNA613441 | SAMN43309870 | Human Stool | IncFIB(S) |
| SAL262 | Salmonella | Enteritidis | 11 | PRJNA613441 | SAMN43309873 | Human Stool | IncFIB(S),IncFII(S) |
| SAL281 | Salmonella | Enteritidis | 11 | PRJNA613442 | SAMN52031898 | Human Stool | IncFIB(S),IncFII(S) |
| SAL282 | Salmonella | Enteritidis | 11 | PRJNA613443 | SAMN52031899 | Human Stool | IncFIB(S), IncFII(S), IncI1-I(Alpha) |
| SAL286 | Salmonella | Enteritidis | 11 | PRJNA613444 | SAMN52031900 | Human Stool | IncFIB(S), IncFII(S) |
| SAL287 | Salmonella | Enteritidis | 11 | PRJNA613445 | SAMN52031901 | Human Stool | IncFIB(S), IncFII(S) |
| SAL293 | *Salmonella* | Enteritidis | 11 | PRJNA613445 | SAMN52031905 | Human Stool | IncFIB(S), IncFII(S) |
| SAL294 | *Salmonella* | Enteritidis | 11 | PRJNA613445 | SAMN52033040 | Human Stool | IncFIB(S), IncFII(S) |
| SAL295 | *Salmonella* | Enteritidis | 11 | PRJNA613445 | SAMN52033041 | Human Stool | IncFIB(S), IncFII(S), IncI1-I(Alpha) |
| SAL271 | Salmonella | Enteritidis | 11 | PRJNA613446 | SAMN43309880 | Human Stool | IncFIB(S),IncFII(S) |
| Resistant_ SAL271 | Salmonella | Enteritidis | 11 | PRJNA1435016 | SAMN56426407 | Phage-resistant derivative of SAL271 | Not Determined |

*Appendix A.2*

**Table A2.1.** Antibiotic Susceptibility Profiles of *Salmonella enterica* Strains Used in This Study part 1.

| ID | Ceftriaxone CRO (R≥ 4) VITEK | Meropenem (R≥ 4) | Ampicillin (R≥ 32) | | Amoxicillin-clavulanic acid -Amoclan (≥8) EUCAST | Gentamicin (R>2) EUCAST | Ciprofloxacin (R≥ 1) |
| --- | --- | --- | --- | --- | --- | --- | --- |
| SAL220 | >64 R | >16 R | <0.25 S | <2 S | | >16 R | 4 R |
| SAL222 | <1 S | <0.25 S | <0.25 S | <2 S | | >16 R | 4 R |
| SAL226 | <1 S | <0.25 S | <0.25 S | <2 S | | >16 R | 4 R |
| SAL227 | <1 S | <0.25 S | <0.25 S | <2 S | | <1 S | 4 R |
| SAL228 | <1 S | <0.25 S | 6 R | 32 R | | >16 R | 4 R |
| SAL236 | <1 S | <0.25 S | <2 S | <2 S | | >16 R | <0.25 S |
| SAL240 | <1 S | <0.25 S | <2 S | <8 S | | <1 S | <0.25 S |
| SAL241 | <1 S | <0.25 S | <2 S | <8 S | | >16 R | <0.25 S |
| SAL242 | <1 S | <0.25 S | <2 S | <8 S | | >16 R | <0.25 S |
| SAL256 | 1 S | 0.25 S | 2 S | 8 R | | >16 R | 0.25 S |
| SAL262 | 1 S | 0.25 S | 2 S | 8 R | | >16 R | >16 R |
| SAL281 | 1 S | 0.25 S | 2 S | 8 R | | >16 R | >16 R |
| SAL282 | 1 S | 0.25 S | 2 S | 8 R | | >16 R | >16 R |
| SAL286 | 1 S | 0.25 S | 2 S | 8 R | | >16 R | >16 R |
| SAL287 | 1 S | 0.25 S | 2 S | 8 R | | >16 R | >16 R |
| SAL293 | S | S | R | R | | R | R |
| SAL294 | S | S | R | S | | R | R |
| SAL295 | R | S | S | R | | R | R |
| SAL271 | 1 S | 0.25 S | 2 S | 8 R | | >16 R | >16 R |

**Table A2.2.** Antibiotic Susceptibility Profiles of *Salmonella enterica* Strains Used in This Study part 2.

| ID | Nalidixic acid (≥32) VITEK | Tetracycline | Chloramphenicol (R ≥ 32) BMD | Colistin (>2) BMD | Trimethoprim-Sulfamethoxazole (≥4/76) VITEK | Azithromycin (≥32) BMD |
| --- | --- | --- | --- | --- | --- | --- |
| SAL220 | 64 R | >16 R | 64 S | 1 S | >512 R | >512 R |
| SAL222 | >512 R | 20 S | 8 S | 8 R | 512 R | 64 R |
| SAL226 | >512 R | >16 R | 64 R | 2 S | >512 R | 64 R |
| SAL227 | >512 R | >16 R | 16 S | 1 S | >512 R | 16 S |
| SAL228 | >512 R | >16 R | 64 R | 1 S | >512 R | 64 R |
| SAL236 | >512 R | <1 S | 64 R | 0.5 S | >512 R | 32 R |
| SAL240 | >32 R | <1 S | 64 R | <0.25 S | >512 R | 2 S |
| SAL241 | >32 R | <1 S | 32 R | <0.25 S | >512 R | 4 S |
| SAL242 | >32 R | <1 S | 16 S | 16 R | <20 S | 2 S |
| SAL256 | 32 R | 16 R | 128 R | 1 S | 16 R | 4 S |
| SAL262 | 32 R | 16 R | 32 R | 0.5 S | 16 R | 4 S |
| SAL281 | 32 R | 16 R | >512 R | <0.25 S | 20 S | 64 R |
| SAL282 | 32 R | 16 R | >512 R | <0.25 S | 20 S | 128 R |
| SAL286 | 32 R | 16 R | >512 R | <0.25 S | 20 S | 64 R |
| SAL287 | 32 R | 16 R | >512 R | <0.25 S | 20 S | 128 R |
| SAL293 | R | R | S | S | R | S |
| SAL294 | R | 16 R | S | S | R | S |
| SAL295 | R | 16 R | S | S | S | S |
| SAL271 | 32 R | 1 S | 256 R | 2 S | 16 R | 4 S |

*Appendix A.3*

**Table A3** Antimicrobial Resistance Profiles of *Salmonella enterica* Strains Used in This Study. Each isolate was classified according to established criteria for multidrug resistance (MDR) and extensively drug-resistant (XDR) phenotypes. MDR is defined as resistance to at least one antimicrobial agent in three or more categories. In contrast, XDR corresponds to resistance to at least one agent in all but two or fewer categories. This table presents the overall resistance classification assigned to each clinical isolate.

| ID | Resistance Profile |
| --- | --- |
| SAL220 | XDR |
| SAL222 | MDR |
| SAL226 | MDR |
| SAL227 | MDR |
| SAL228 | MDR |
| SAL236 | MDR |
| SAL240 | MDR |
| SAL241 | MDR |
| SAL242 | MDR |
| SAL256 | MDR |
| SAL262 | MDR |
| SAL281 | MDR |
| SAL282 | MDR |
| SAL286 | MDR |
| SAL287 | MDR |
| SAL293 | MDR |
| SAL294 | MDR |
| SAL295 | MDR |
| SAL271 | MDR |

**Supplementary Table 1.** Summary of Genome safety screening of isolated Salmonella Phage GF04.

| Phage ID | Integrase | Excisionase | Site-specific recombinase | CI-like repressor | Virulence genes | Antimicrobial resistance (AMR) genes |
| --- | --- | --- | --- | --- | --- | --- |
| GF04 | Not detected | Not detected | Not detected | Not detected | Not detected | Not detected |
